# Letter to the Editor of European Archives of Otorhinolaryngology about a paper “Classification of parotidectomies: a proposal of the European Salivary Gland Society” by Quer et al.

**DOI:** 10.1007/s00405-016-4014-5

**Published:** 2016-04-06

**Authors:** Małgorzata Wierzbicka

**Affiliations:** Department of Otolaryngology and Laryngological Oncology, University of Medical Sciences, Przybyszewskiego 39, 60-663 Poznan, Poland

Dear Editor,

A paper ‘’Classification of parotidectomies: a proposal of the European Salivary Gland Society” by M. Quer et al. recently published in this journal provides comprehensive classification of the parotidectomy.

The first note worthy contribution unifies the surgical anatomy of the parotid region. Although during the embryogenesis the parenchyma is evolved as one indivisible whole anatomic. A surgical division of the parotid gland to two or three parts has been the practical norm. The relation between the parotid gland, facial nerve and the cover plans suggest bilobular architecture or three ‘’lobes’’ in relation to facial nerve main branches. The ESGS proposes to accept and use the Barcelona classification with one modification. Finally the division of the parotid gland into five levels has been adopted by ESGS to report the surgery performed.

The key issue that was approved was the classification system for parotid surgery, which uses two basic terms to define the procedures: extracapsular dissection and parotidectomy. In all descriptions of the operation the number levels and nonparotid structures are specified.

The simple, clear and comprehensive classification is especially valuable for centers, that lead registration. In our institution the Poland National Registry of Benign Parotid Tumors has been conducted and a variety of techniques, along with certain dispersion in the criteria that define them, has led to some confusion about the surgery performed. Thus we are personally grateful for this new classification, that facilitates multicentre communication.

I would like to take this opportunity if I may to pose one question. The temporomandibular joint (TMJ) is in the immediate vicinity of the I and IV parotid levels [1, 2]. This structure may require surgical violation or resection, not only when involved in primary tumours of the bone adjacent to the mandible, but in tumours derived from the parotic [3, 4]. TMJ is also important in using joint structures as margins in tumour resection. In Table 5 non-parotic structures that could be removed are listed. I wonder if besides the mastoid bone and lateral temporal resection, additional TMJ should be included, although it could possibly be placed in category “others to be defined”.
